# Decoding Global Palates: Unveiling Cross-Cultural Flavor Preferences Through Online Recipes

**DOI:** 10.3390/foods14081411

**Published:** 2025-04-18

**Authors:** Qing Zhang, David Elsweiler, Christoph Trattner

**Affiliations:** 1 School of Information Management, Sun Yat-sen University, Guangzhou 510006, China; 2 Institute for Language, Literature and Culture, University of Regensburg, 93053 Regensburg, Germany; david.elsweiler@ur.de; 3 MediaFutures, University of Bergen, 5008 Bergen, Norway; christoph.trattner@uib.no

**Keywords:** food preferences, flavor compounds, food cultures, food recommender systems

## Abstract

Navigating cross-cultural food choices is complex, influenced by cultural nuances and various factors, with flavor playing a crucial role. Understanding cultural flavor preferences helps individuals make informed food choices in cross-cultural contexts. We examined flavor differences across China, the US, and Germany, as well as consistent flavor preference patterns using online recipes from prominent recipe portals. Distinct from applying traditional food pairing theory, we directly mapped ingredients to their individual flavor compounds using an authorized database. This allowed us to analyze cultural flavor preferences at the molecular level and conduct machine learning experiments on 25,000 recipes from each culture to reveal flavor-based distinctions. The classifier, trained on these flavor compounds, achieved 77% accuracy in discriminating recipes by country in a three-class classification task, where random choice would yield 33.3% accuracy. Additionally, using user interaction data on appreciation metrics from each recipe portal (e.g., recipe ratings), we selected the top 10% and bottom 10% of recipes as proxies for appreciated and less appreciated recipes, respectively. Models trained within each portal discriminated between the two groups, reaching a maximum accuracy of 66%, while random selection would result in a baseline accuracy of 50%. We also explored cross-cultural preferences by applying classifiers trained on one culture to recipes from other cultures. While the cross-cultural performance was modest (specifically, a max accuracy of 54% was obtained when predicting food preferences ofthe USusers with models trained on the Chinesedata), the results indicate potential shared flavor patterns, especially between Chinese and US recipes, which show similarities, while German preferences differ. Exploratory analyses further validated these findings: we constructed ingredient networks based on co-occurrence relationships to label recipes as savory or sweet, and clustered the flavor profiles of compounds as sweet or non-sweet. These analyses showed opposing trends in sweet vs. non-sweet/savory appreciation between US and German users, supporting the machine learning results. Although our findings are likely to be influenced by biases in online data sources and the limitations of data-driven methods, they may still highlight meaningful cultural differences and shared flavor preferences. These insights offer potential for developing food recommender systems that cater to cross-cultural contexts.

## 1. Introduction

Deciding what to eat is a complex process shaped by many contextual factors [1,2,3,4], among which culture plays a significant role [5,6,7,8]. Varied cultural and regional groups have different eating habits [9,10,11,12,13]. Individuals commonly find it is easier to make food choices within their familiar cultural contexts due to a sense of identity and familiarity [14,15,16], while in cross-cultural scenarios such as travel or migration, they often navigate between exploring new cuisines and maintaining ties to their own food culture [17,18], adding further complexity to food decision-making. Food recommender systems have emerged to help users manage the overwhelming number of food options by tailoring suggestions to personal preferences. Previous work has also emphasized the importance of incorporating cultural elements into recommendation systems [19]. Nevertheless, understanding cross-cultural flavor preferences remains an ongoing challenge.

Traditional anthropological methods, such as case studies and questionnaires, have provided valuable insights into global dietary patterns. By focusing on small groups within specific ethnic communities, anthropologists have not only uncovered the unique characteristics of each culture [20,21,22], but also explored how various factors—such as environmental contexts [14,23,24,25,26], rituals [1,27,28], religions [29,30,31], and aesthetic ideals [32,33,34,35,36]—shape food practices, either individually or in combination. However, in-depth qualitative approaches are expensive and time-consuming. Over the past decade, online recipe portals have increasingly become a testbed for studying cross-cultural dietary patterns. By applying computational methods on large-scale online sources, such as online recipes, and users’ activity on social media, researchers can identify broad differences in food preferences with quantitative evidence. For example, research leveraging large-scale recipe datasets demonstrated significant variations in food choices across cultures. Kim and Chung [12] found that recipes from 22 countries feature distinct ingredients, such as ginger and soy sauce in Asian recipes and olive oil in European cuisines. Similarly, Sajadmanesh et al. [37] found that user-generated recipes from 200 countries have unique ingredient patterns, allowing recipes from different cultures to be classified algorithmically. Despite these differences, previous research has also identified similarities. Studies suggest a global balance between complexity and simplicity in dietary patterns, with the average number of ingredients typically ranging from 8 to 11 across cultures [9,38,39]. In addition to the cross-cultural differences and commonalities revealed in the ingredients, our previous research has also analyzed the sensory component, particularly the visual appearance of food [40,41]. These works suggest that both differences and stable patterns exist across cultures, which should be considered when developing food recommender systems. Building on this finding, the current work focuses on the other sensory input of food, flavor.

Flavor plays a significant role in food decision-making, while cultural factors shape flavor preferences. Laboratory experiments and surveys examining samples from various ethnic groups have demonstrated distinct sensitivities to tastes [42,43]. Recent studies incorporating methodologies from the domain of computational gastronomy [44] have also highlighted cross-cultural flavor preferences. For example, a notable food pairing theory has been raised based on the co-occurrence of flavor compounds encoded in the ingredients. This theory posits that ingredients sharing flavor compounds are more likely to taste better together than ingredients that do not. Research found that recipes originating from Western countries often adhere to this theory, while Eastern cuisines deviate from it. Similar investigations have been extended to exploring specific cuisines from various regions, including India [45], Arabia [46] and China [47], validating the applicability of the food pairing theory. These works have been facilitated partly due to the availability of databases containing food and chemical flavor information, such as “*Fenaroli’s Handbook of Flavor ingredients*” [48] and the recently released FlavorDB [49]. Despite its contributions, the food pairing framework offers a limited perspective by focusing mainly on pairwise ingredient relationships. The literature has focused less attention on how flavor compounds can represent recipes or how they relate to user preferences and cultural appreciation. Our work addresses this gap by exploring alternative ways to use flavor information in understanding cross-cultural flavor patterns. Specifically, we represent recipes through their underlying flavor compounds and apply data-driven approaches, including machine learning classification tasks and exploratory analyses, to examine cultural differences and commonalities in flavor preferences.

In this work, we study flavor differences and stable patterns in food preferences across geographically distinct regions—Asia, North America, and Europe—by examining three cultures: China, the US, and Germany. These countries were selected not only for their distinct culinary traditions but also to maintain continuity with our previous work [40,41], which explored the visual appearance of food across the same cultures. By focusing on flavor, another key aesthetic dimension, we ensure comparability while aiming to improve food recommender systems in cross-cultural contexts.

We represented recipes with flavor compounds encoded in the ingredients. By applying machine learning algorithms, we trained classifiers to differentiate the recipes from the recipe portals of China, the US, and Germany. We continued to identify food preferences within each culture using the same machine learning approaches on appreciated and less appreciated recipes from each portal. In order to examine stable patterns in cross-cultural flavor preferences, we applied classifiers trained on one recipe portal to other portals. The performance of the classifiers trained with flavor compounds would provide insight into understanding cross-cultural flavor preferences. Additionally, we used models trained with ingredients as a baseline to evaluate the ability of flavor compounds to distinguish recipe origins and predict food preferences. This approach was motivated by the established effectiveness of ingredients in distinguishing cuisines, with classifiers trained on ingredients consistently achieving good accuracy in cuisine prediction tasks [12,37]. To complement the machine learning experiments, we conducted several additional exploratory analyses. We built flavor description systems utilizing the flavor information, specifically to label flavors as savory/non-sweet and sweet. We then calculated the proportion of savory/non-sweet and sweet recipes in appreciated and less appreciated recipes in each portal. This aimed to substantiate our findings by revealing possible explanations for the found trends.

Building on the research described above, we extend the state of the art in two main ways. First, we develop a more comprehensive understanding of the sensory dimensions of food preferences across distinct cultures with flavor information, extending beyond traditional ingredient-based analyses with the large-scale online recipes. Second, in previous work, studies associated with cross-cultural flavor preferences have heavily relied on the food pairing theory. We, instead, explore alternative roles of flavor information in identifying broader cross-cultural dietary patterns. The findings of this study offer a new perspective for developing a cross-cultural food recommender by exploring both differences and commonalities in flavor preferences. These insights could help inform recommendation algorithms that are better aligned with local flavor expectations in cross-cultural contexts.

## 2. Materials and Methods

**Figure 1:**
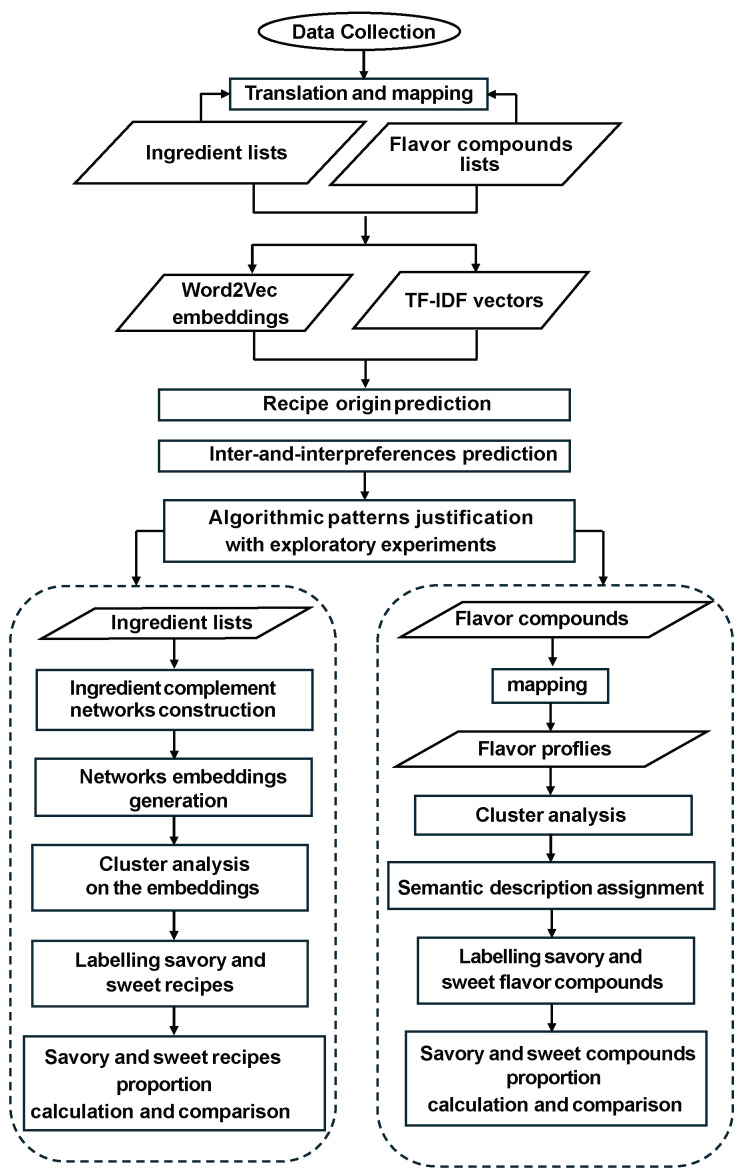
Flowchart of experiments conducted in this work.

In this section, we describe the data sources used in our study and outline the methods applied throughout the analysis. To facilitate understanding, we also present a flowchart in Figure 1 that visually summarizes the experimental workflow employed in this work.

### 2.1. Data Collection

In this work, we obtained data from the online recipe portals *Xiachufang* (China), *Allrecipes* (the US), and *Kochbar* (Germany), and applied online recipe preferences as a proxy of population-level dietary preferences. Our work builds upon previous work that demonstrated that such data closely reflect individuals’ real-world consumption patterns. For example, strong correlations between interaction patterns with *Allrecipes* recipes and obesity rates in different US geographical regions were revealed in both [50,51]. In [52], the authors also identified correlations between sodium levels in recipes over time and patterns of admission for congestive heart failure, a chronic condition that can be aggravated by high sodium intake. Similar trends were also observed by using data collected via the German online food portal *Kochbar* [38]. In this work, we focus on cross-cultural food preferences, deriving data from three large and well-known recipe portals—*Xiachufang* (N = 25,597), *Allrecipes* (N = 35,501), and *Kochbar* (N = 72,899), from China, the US, and Germany, respectively. The data from *Allrecipes* and *Kochbar* were gathered in 2014 and 2015, respectively, when the websites allowed data to be scraped using standard web crawlers [53]. The Chinese recipes were crawled from *Xiachufang* between 22 and 26 October 2018. However, this dataset includes recipes uploaded during the same period as those collected from the other two portals. We adhered to the terms and conditions when scraping data from these platforms. Although the online recipes and corresponding interaction data were scraped a few years ago, they have still been actively used for investigating dietary patterns in recent studies [54,55], suggesting their representativeness and reliability. Several key factors informed our decision to source data from these specific portals: First, these portals are consistently ranked among the top five national websites in the category of *Cooking and Recipes*. This indicates that they host an abundance of user-generated online recipes and interaction data suitable for our research. Secondly, these platforms provide recipes in Chinese, English, and German, corresponding to their respective regions. According to Similarweb, the majority of users for each platform reside in the corresponding country. To be specific, 62.81% of *Xiachufang* users are from China, 81.17% of *Allrecipes* users are from the US, and 84.92% of *Kochbar* users are from Germany [56]. We therefore assume that the majority of users are native speakers, making the recipes culturally authentic and representative. Furthermore, these recipe portals encourage users to upload home-cooked recipes, which, to some extent, provide insight into the types of meals that people commonly prepare and eat. While this doesn’t guarantee full representativeness, it offers a broader spectrum of culinary and dietary preferences. Previous research [53,57,58,59], including our own studies [40,41], has confirmed the reliability of these platforms for studying digital food preferences.

We selected 25,000 recipes from each portal to analyze flavor differences across cultures, which is approximately the total number of recipes available from *Xiachufang*. To ensure a balanced dataset for the prediction task applying machine learning approaches, we randomly sampled 25,000 recipes from each collection, minimizing potential biases in the analysis. These samples were also re-used from our previous work [40]. To investigate flavor preferences within each culture, we established several binary classification tasks. Each task was conducted using 2500 recipes from the top 10% (appreciated) and 2500 recipes from the bottom 10% (less appreciated) based on the appreciation metrics available on each recipe portal. For *Xiachufang*, user ratings were used as the appreciation metric, as this is the only form of interaction data available on the platform. For *Allrecipes* and *Kochbar*, we adopted alternative metrics, such as the count of bookmarks a recipe received within the first year since it had been uploaded, and the frequency with which recipes were marked as favorites, as recommended in previous research [53]. These alternatives were selected because the rating distributions on these two platforms are highly skewed, with low variance, limiting their effectiveness as preference indicators [53]. In addition, the appreciated and less appreciated samples were also applied in our previous work on cross-cultural visual food preferences [41]. We therefore retained the same samples in this study to ensure consistency and comparability across our examination of different aesthetic dimensions of food, namely visual appearance and flavor.

For the Chinese and German ingredients, we first translated them into English using the Google Cloud Translation API. To ensure accuracy and minimize potential cultural or linguistic biases, we then manually reviewed and mapped the translated ingredients in the *Allrecipes* collection to the corresponding entity names in FlavorDB [49], a curated database containing 936 ingredient entities and their associated 2254 flavor molecules. This mapping process allowed us to standardize ingredient representation across cultures. Finally, we linked each ingredient to its associated flavor compounds using the FlavorDB taxonomy. The full data preparation workflow—including data cleaning, translation, mapping, and filtering—is detailed in Appendix A.1.

### 2.2. Recipe Representation and Model Training

We represented each recipe using ingredient and flavor compound vectors generated through TF-IDF and Word2Vec. For Word2Vec, we applied the CBOW algorithm, where each ingredient or flavor compound was embedded based on its surrounding context within the recipe. To reduce noise caused by arbitrary ordering, we sorted the ingredients and flavor compounds within each recipe alphabetically before training. This approach was recommended by Rita et al. [60] as a strategy for handling unordered ingredient data. The word embeddings were trained using the *Gensim 4.1.2* library in Python 3.9 [61]. For the dimension of the embeddings, we used a default value of 128. For parameters like *num_walk* and *walk_length*, we tested various values and used UMAP to visualize and select the most effective settings. We set *p* = 2 to reduce revisits and *q* = 0.25 to encourage deeper exploration of the ingredient networks. We show the fine-tuning of the hyperparameters of Word2Vec in Appendix A.2 with more details. After obtaining the representations of each ingredient and flavor compound, we computed a recipe-level representation by averaging all the embeddings for the ingredients and flavor compounds in each recipe.

We employed three classification algorithms, including Naïve Bayes (NB), Logistic Regression (LOG), and Random Forest (RF), to train models for both recipe origin identification and recipe preference prediction tasks. These algorithms were chosen to ensure methodological consistency with our previous studies on cross-cultural visual food biases and preferences, thereby enabling comparability across different aesthetic aspects of food. The optimal parameters for LOG and RF were determined using 5-fold randomized search cross-validation. As a first step, we trained the models on the dataset of 75,000 recipes (25,000 from each portal) to evaluate the effectiveness of ingredient and flavor compound representations in distinguishing recipes by their portal of origin. Classifier performance was evaluated using accuracy (ACC), and misclassifications were visualized using a confusion matrix.

We then employed the same algorithms to train classifiers for discriminating appreciated and less appreciated recipes within each portal, with ACC serving as the performance metric. In the next step, we tested the predictive ability of the best-performing classifier trained on each recipe portal by applying it to the other two portals. This approach allowed us to examine whether flavor preferences learned in one cultural context could be transferred to others. The same approach was also employed in our previous work, which revealed stable patterns in cross-cultural food preferences from the visual aspects [41]. We extended this approach to examine the potential for identifying cross-cultural flavor preferences.

### 2.3. Design of the Exploratory Analyses

In this section, we describe two exploratory analyses utilizing ingredients and their encoded flavor information. Our aim is to supplement the findings from the machine learning experiments by identifying potential explanations for the patterns identified by the algorithms.

#### 2.3.1. Identifying Savory and Sweet Recipes by Means of Ingredient Complement Networks

This analysis is inspired by the previous work [62], in which the authors built an ingredient complement network based on the co-occurrences of ingredients. In that work, ingredient complementarity is inferred from cultural and behavioral patterns in recipe construction rather than from direct sensory validation. Previous research has shown that such networks tend to form distinct subcommunities, such as those associated with sweet or savory dishes. Following this approach, we built our networks based on co-occurrence data from online recipe portals. We then categorized recipes as either savory or sweet using a node embedding algorithm (Node2Vec) and an unsupervised learning approach (K-means clustering). Subsequently, we calculated the proportion of savory/sweet recipes in appreciated and less appreciated recipes in each collection, respectively. By doing this, we intended to identify the preferences for savory and sweet tastes in the Chinese, US, and German cultures. The steps of the analysis are described as follows:

**Step 1: Generating ingredient complement networks**

This network was generated based on the complementary relationship between the ingredients of each recipe collection. Each node denotes an ingredient, and two nodes share an edge if the likelihood of these two ingredients occurring in one recipe exceeds a threshold, which is defined by the pointwise mutual information (*PMI*). *PMI* indicates the probability of a pair of ingredients co-occurring *p*(*a*,*b*) against the probability that they occur separately *p*(*a*), *p*(*b*):

(1) $$PMI\left(a,b\right)=\log\frac{p(a,b)}{p(a)p(b)}$$

where

(2) $$\begin{array}{c} p\left(a,b\right)=\frac{\#\;\mathrm{of}\;\mathrm{recipe}\;\mathrm{where}\;a\;\mathrm{and}\;b\;\mathrm{co-occur}}{\#\;\mathrm{of}\;\mathrm{recipes}}\\ p\left(a\right)=\frac{\#\;\mathrm{of}\;\mathrm{recipes}\;\mathrm{where}\;a\;\mathrm{occurs}}{\#\;\mathrm{of}\;\mathrm{recipes}}\\ p\left(b\right)=\frac{\#\;\mathrm{of}\;\mathrm{recipes}\;\mathrm{where}\;b\;\mathrm{occurs}}{\#\;\mathrm{of}\;\mathrm{recipes}} \end{array}$$

*PMI* emphasizes the complementary ingredients, which tend to occur together far more often than would be expected by chance. We normalized the *PMI* (*NPMI*), as in [63], in order to limit its range to between −1 and 1, where −1 means that a pair of ingredients would never occur together, and 1 indicates they would definitely co-occur. *NPMI* is measured as follows:

(3) $$NPMI\left(a,b\right)=\frac{PMI(a,b)}{h(a,b)}$$

where

(4) h(a,b)=−logp(a,b)

To generate the complement networks containing highly complementary ingredients in the network, we set a threshold to select the candidate ingredients and ingredient pairs. Initially, we selected ingredients that appeared more than 10 times individually, and pairs of ingredients that appeared together more than 5 times. Additionally, we retained ingredient pairs with an *NPMI* exceeding 0.10 to generate the complementary network. This threshold was determined based on the median *NPMI* value across all three recipe collections, which was approximately 0.10. By applying this threshold, we aimed to display highly complementary ingredients in the network while maintaining representativeness in our exploratory analysis.

**Step 2: Representing ingredients and recipes using Node2Vec**

After establishing the ingredient complement networks, we represented their nodes with embeddings using Node2Vec [64], which maps the nodes in a network to a feature space while preserving the initial structure of the network. We show the details of fine-tuning the parameters in Appendix B.

After representing each ingredient in the complement network, we employed these embeddings to create recipe representations. This process resembles the method we applied to generate recipe representation with the word embeddings of ingredients/flavor compounds using Word2Vec. In both cases, we calculated the representation for each recipe by summing up all ingredient embeddings and dividing them by the length of the recipes.

**Step 3: K-means on the recipe representation**

In this step, we applied K-means on the recipe representation. We set the value of *k* to 2 according to the previous study [62], in which there are two subcommunities shown in the ingredient complement network, with the aim being to categorize the recipes as savory or sweet. For each cluster of recipes, we weighted the ingredients by their frequency of occurrence and visualized them in word clouds. This helped us to identify the clusters.

**Step 4: Calculating the proportion of savory/sweet recipes in appreciated and less appreciated recipes**

Steps 1–3 offer a system to label recipes as either savory or sweet. To quantify the differences and similarities in flavor preferences across cultures, we calculated and compared the proportions of savory and sweet recipes within both the appreciated and less appreciated categories of each recipe collection.

#### 2.3.2. Building Semantic Clusters for Flavor Compounds Based on Their Corresponding Flavor Profiles

**Figure 2:**
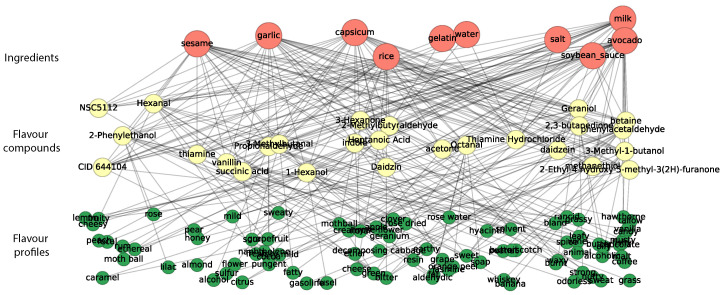
Relation network of *<ingredient–flavor compounds>* and *<flavor compounds–flavor profiles>*.

We constructed a flavor description system using the flavor profiles of the flavor compounds. In FlavorDB, the flavor compounds have a list of flavor profiles, which describe the flavor compounds semantically with a series of words. We display the relationship between *<ingredient–flavor compounds>* and *<flavor compounds–flavor profile>* in Figure 2.

The basic idea of this analysis was to cluster the flavor compounds with similar flavor profiles into one group and assign semantic descriptions for each cluster based on these profiles. Subsequently, we analyzed the proportions of each cluster of flavor compounds within both the appreciated and less appreciated recipes in each recipe collection. These proportions were then compared across recipe collections to determine whether they reveal similar or different trends in flavor preferences across cultures. The entire process is detailed below:

**Step 1: Representing flavor compounds with their corresponding flavor profiles**

For each flavor compound that occurred more than three times in each recipe collection and had corresponding flavor profiles (N = 927), we transformed them into Boolean bag-of-flavor-profile vectors. These vectors indicate the presence of a flavor profile using Boolean values, where 0 represents absence, and 1 signifies presence. Consequently, each flavor compound was depicted as a vector with a length matching the total number of flavor profiles, which is 510.

**Step 2: K-means on the representation of flavor compounds**

**Figure 3:**
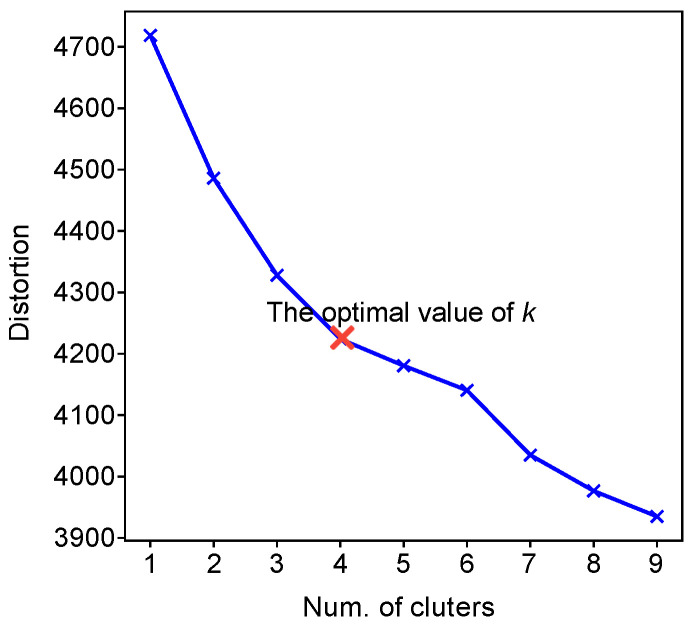
The elbow method for determining the optimal *k*. Four should be the optimal number of clusters for the flavor compounds.

The K-means algorithm was applied in this step to partition flavor compounds with similar flavor profiles into *k* distinct clusters. In order to determine the optimal number of clusters, i.e., the value of *k*, we applied the elbow method, which plots the value of the cost function produced by a different value of *k* (ranging from 1 to 9 in this study). The optimal *k* lies on the point (i.e., the elbow) where the value of the cost function becomes relatively flat after it decreases sharply. The elbow method determined the optimal value of *k* to be 4, as shown in Figure 3.

**Step 3: Assigning semantic description for each cluster**

After determining the number of clusters for the flavor compounds, we assigned a semantic description for each cluster based on the representative of their flavor profiles. To be specific, for one of the flavor compound clusters, we first measured the prevalence of a flavor profile *p* for describing the flavor compounds in cluster *c* as follows:

(5) $${P_{p}}^{c}=\frac{{n_{p}}^{c}}{N_{c}}$$

where ${n_{p}}^{c}$ is the prevalence of *p* for describing flavor compounds in cluster *c*, and $N_{c}$ is the total number of flavor compounds in cluster *c*.

Then, the representativeness of the flavor profiles in cluster *c* was measured with the formula

(6) $$P_{p}^{c}=P_{p}^{c}-{\langle P_{p}^{c^{\prime }}\rangle }_{c^{\prime }\neq c}$$

where $\langle P_{p}^{c^{\prime }}\rangle$ is the average prevalence of the profile *p* for describing the flavor compounds in other clusters except *c*. This shows the difference between the prevalence of a profile *p* for describing the flavor compounds in one cluster *c* and the average prevalence of *p* for describing the flavor compounds in other clusters. These formulas have been previously utilized in [9] to assess the authenticity of ingredients in different cuisines. In this study, we employed these formulas to identify the most representative flavor profiles for the flavor compounds in each cluster. Finally, we visualized them in word clouds.

**Step 4: Calculating the ratio of each cluster of flavor compounds in appreciated and less appreciated recipes**

In the following step, we calculated the ratio of each cluster of flavor compounds in both the appreciated and less appreciated recipes from each recipe collection to investigate flavor preferences. Since flavor compounds are intricately associated with ingredients, we determined these ratios based on the ingredients. Instead of considering all of the ingredients in the recipe collections, we opted to focus on distinctive ingredients. This choice is influenced by previous work [65], in which the authors indicated that it is possible to represent regional cuisines with a few key ingredients, such as soybean sauce in Chinese recipes, and paprika, onion, and lard in Hungarian cuisine [9]. We took this into account and used distinctive ingredients to represent the appreciated and less appreciated recipes in each collection. We attributed the distinctive ingredients in appreciated recipes to those that appeared more frequently in appreciated recipes than less appreciated ones. To identify these ingredients, we applied the weighted log odds ratio [66], calculated as follows:

(7) $$\log\;\mathrm{odds}\;\mathrm{ratio}=\ln(\frac{{\left[\frac{n+1}{total+1}\right]}_{\mathrm{Appreciated}\;\mathrm{Recipes}}}{{\left[\frac{n+1}{total+1}\right]}_{\mathrm{Less}\;\mathrm{Appreciated}\;\mathrm{Recipes}}})$$

where *n* is the frequency of an ingredient in both appreciated and less appreciated recipes, while *total* indicates the total number of ingredients in both categories. Ingredients with a higher odds ratio are considered more distinctive in the recipe collections. We selected the top 50 ingredients based on their weighted log odds values as the distinctive ingredients in the appreciated and less appreciated recipes for each recipe collection. After identifying these distinctive ingredients, we calculated the ratio of each cluster of flavor compounds in the appreciated and less appreciated recipes. This allowed us to validate the patterns revealed by the algorithms regarding flavor preferences across different cultures.

## 3. Results

In this section, we present the outcomes of the cross-cultural categorization tasks for food flavor preferences, along with exploratory analyses aimed at validating the findings.

### 3.1. Recipe Origin Prediction with Ingredients and Flavor Compounds

Table 1 illustrates the performance of the classifiers trained using ingredients and flavor compound vectors for classifying the origin portals of the recipes.

Overall, all classifiers, for both ingredients and flavor compounds, demonstrate reasonable accuracy in discriminating recipes sourced from China, the US, and German recipe portals, especially when recipes are represented using TF-IDF weightings. The accuracy (ACC) values of classifiers trained with TF-IDF ingredient and flavor compound representations are 81% and 77%, respectively. Discriminating recipes’ origin portals is a three-class classification problem, where a random baseline would yield an ACC of 33.3%. Our classifiers significantly outperform this baseline. This indicates that recipes from different cultures exhibit significant distinctions in both ingredient usage and flavor, allowing for effective algorithmic differentiation.

Classifiers trained on ingredient representations outperformed those using flavor compounds, suggesting greater similarity in flavor across cultures. These results align with previous studies indicating that ingredients are more discriminative than flavor [37]. Additionally, TF-IDF vectors consistently outperformed Word2Vec embeddings. This finding suggests that word-frequency-based approaches might be better suited for recipe classification tasks than word embedding techniques. However, this may be partially attributed to limitations in the Word2Vec training process (e.g., vocabulary size and preprocessing), rather than an inherent superiority of the method. Future work using larger and more diverse datasets would be beneficial to better evaluate the effectiveness of Word2Vec in capturing ingredient-level or flavor-related representations.

**Figure 4:**
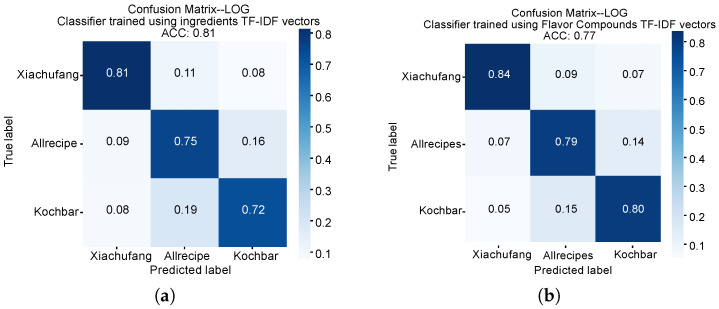
The confusion matrices of the classifiers for discriminating recipes from *Xiachufang*, *Allrecipes*, and *Kochbar*. (**a**) The classifier trained using ingredient TF-IDF vectors. (**b**) The classifier trained using flavor compound TF-IDF vectors.

We also observed that classifiers performed better in identifying recipes from the Chinese recipe portal with ingredient and flavor representations (ACC = 81% and 84%) compared to the US (ACC = 75% and 79%) and German portals (ACC = 72% and 80%) (Figure 4). This finding suggests that recipes from the US and Germany exhibit greater similarity in terms of ingredient usage and flavor, while Chinese recipes appear more distinct in terms of both ingredient usage and flavor. This corresponds with our previous work on visual aspects [40], which found Chinese recipes to be the most visually distinct, while those from the US and German portals were more likely to be confused with each other.

### 3.2. Identifying Intra- and Inter-Cultural Flavor Preferences with Machine Learning Approaches

The same three algorithms were trained on the appreciated and less appreciated recipe samples from each recipe portal. The performance (51% < ACC < 70%) shown in Table 2 indicates that classifiers trained using flavor compound vectors are able to differentiate appreciated and less appreciated recipes within each culture. It is also found that flavor information shows comparable predictive efficacy to ingredients in this task. Preferences prediction is a binary classification task, where the random baseline is 50%. The ACC of our models still indicates meaningful predictive power. However, predicting preferences proves to be more challenging compared to classifying recipe sources (ACC = 77% vs. avg ACC = 66%). Additionally, TF-IDF once again outperformed Word2Vec in our experiments.

**Figure 5:**
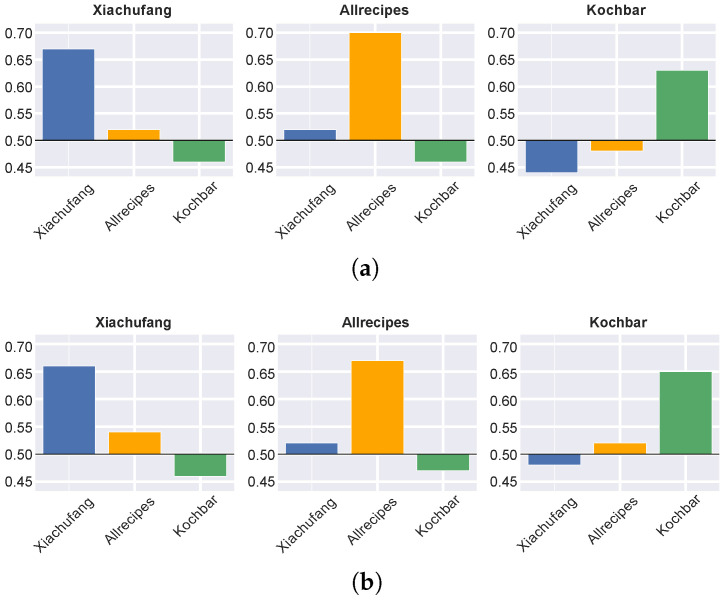
The best-performing models for predicting food preferences within each culture and their performance on the other two portals. (**a**) The classifier trained using ingredient TF-IDF vectors. (**b**) The classifier trained using flavor compound TF-IDF vectors.

After assessing the predictive abilities of ingredients and flavor compounds for food preferences within each culture, we chose the top-performing classifier for each recipe collection. These classifiers, trained with TF-IDF vectors, were then tested for their effectiveness in differentiating appreciated and less appreciated recipes in the remaining two collections. We display the results in Figure 5a,b. We observed that classifiers trained on *Xiachufang* and *Allrecipes* recipes performed well on their own collections and moderately well on each others’ collections, achieving an accuracy higher than 50% in all cases. To be specific, when applying the flavor compounds, the classifier trained on *Xiachufang* achieved an ACC of 54% in predicting the preferences of *Allrecipe* users. Additionally, the *Allrecipes* classifier achieved 52% in predicting *Xiachufang* user preferences. However, they both performed very poorly on *Kochbar* recipes. The classifiers trained on recipes from *Kochbar*, in contrast, showed limited generalizability. Specifically, the classifier trained using ingredients of the German recipes only performed well on the *Kochbar* collection (ACC = 64%), but its performance was poor when applied to *Xiachufang* (ACC = 44%) and *Allrecipes* (ACC = 48%) recipes. While the *Kochbar* classifier trained using the flavor compounds performed slightly better on *Allrecipes* recipes (ACC = 52%), it still performed very poorly on *Xiachufang* recipes (ACC = 48%). These results suggest that Chinese and US preferences are more similar in terms of ingredient usage and flavor than German preferences. This finding challenges previous findings [37], which indicate that flavor preferences in Western countries are more similar to each other than to those in Oriental cultures. Furthermore, the observed trends also differ from those in our previous work, in which the US and German recipes demonstrate greater similarity, while Chinese visual preferences stand out as the outlier [41]. In order to justify the patterns revealed by applying machine learning approaches, we designed and conducted a series of data-driven exploratory analyses in the next step.

By applying machine learning algorithms, we observed stable patterns of flavor preference across cultures, particularly between China and the US. However, this finding contrasts with earlier research, making it an unexpected result. Our following exploratory analyses were conducted to elucidate the patterns unveiled by the algorithms.

### 3.3. Exploratory Analyses Justified the Discovered Patterns in Cross-Cultural Flavor Preferences

In our two exploratory analyses, we developed two description systems of flavors to elucidate cross-cultural flavor preferences. These partially explain and support the patterns observed in the prediction experiments.

#### 3.3.1. Preferences for Savory and Sweet Recipes Across Cultures

**Figure 6:**
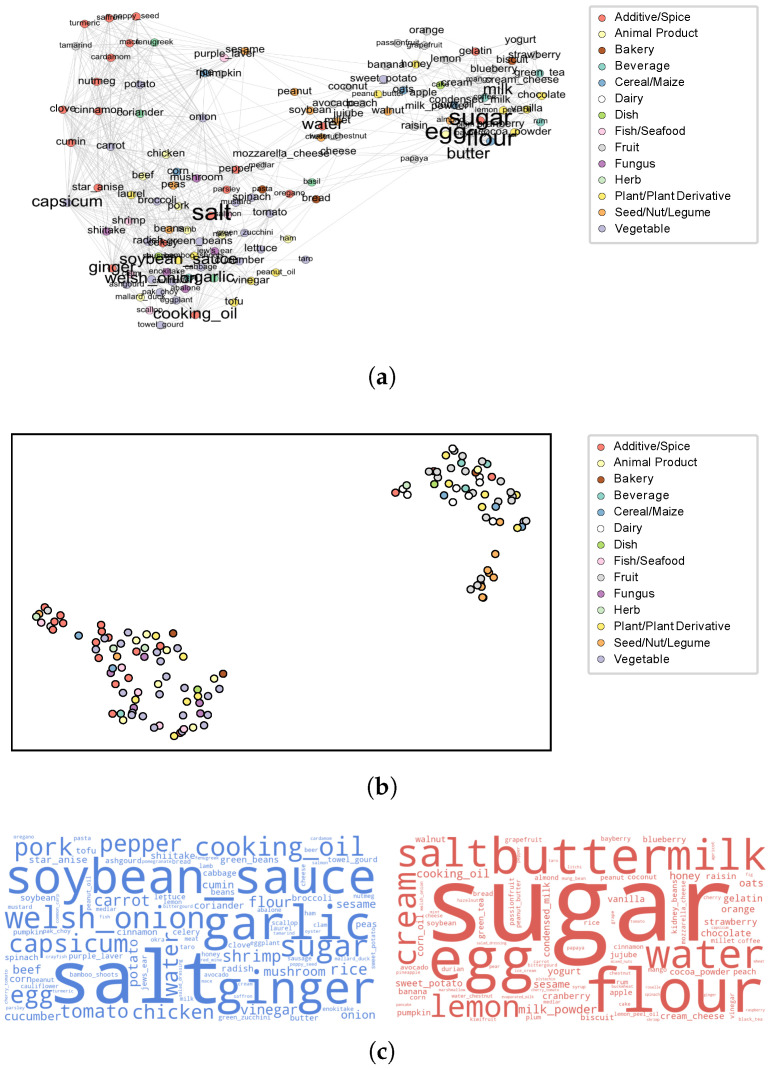
Subcommunities of savory/sweet ingredients in appreciated and less appreciated recipes from *Xiachufang*. (**a**) Ingredient complement network of appreciated and less appreciated recipes from *Xiachufang*. (**b**) UMAP of Node2Vec embeddings generated from the network. (**c**) Word clouds of savory (**left**) and sweet (**right**) recipe ingredients in appreciated and less appreciated recipes from *Xiachufang*. For the ingredient complement networks and the corresponding UMAP and word clouds of recipes from *Allrecipes* and *Kochbar*, please see Appendix C and Figure A1 and Figure A2.

**Figure A1:**
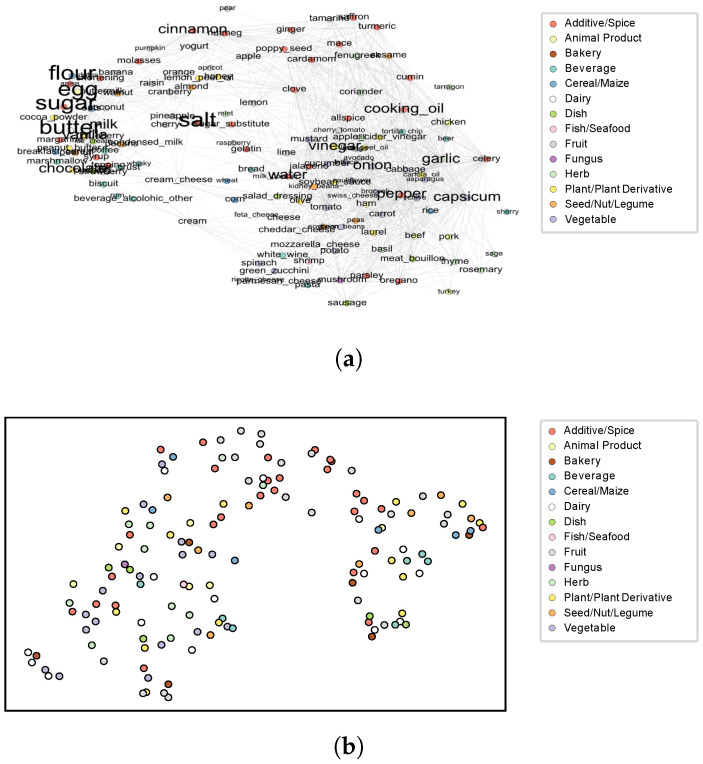

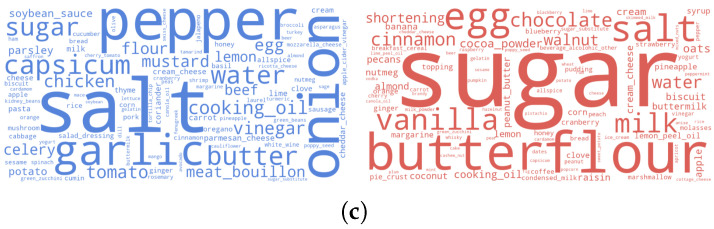
Subcommunities of savory/sweet recipes in appreciated and less appreciated recipes from *Allrecipes*. (**a**) Ingredient complement network of appreciated and less appreciated recipes from *Allrecipes*. (**b**) UMAP of Node2Vec embeddings generated from the network. (**c**) Word clouds of savory (**left**) and sweet (**right**) recipe ingredients in appreciated and less appreciated recipes from *Allrecipes*.

**Figure A2:**
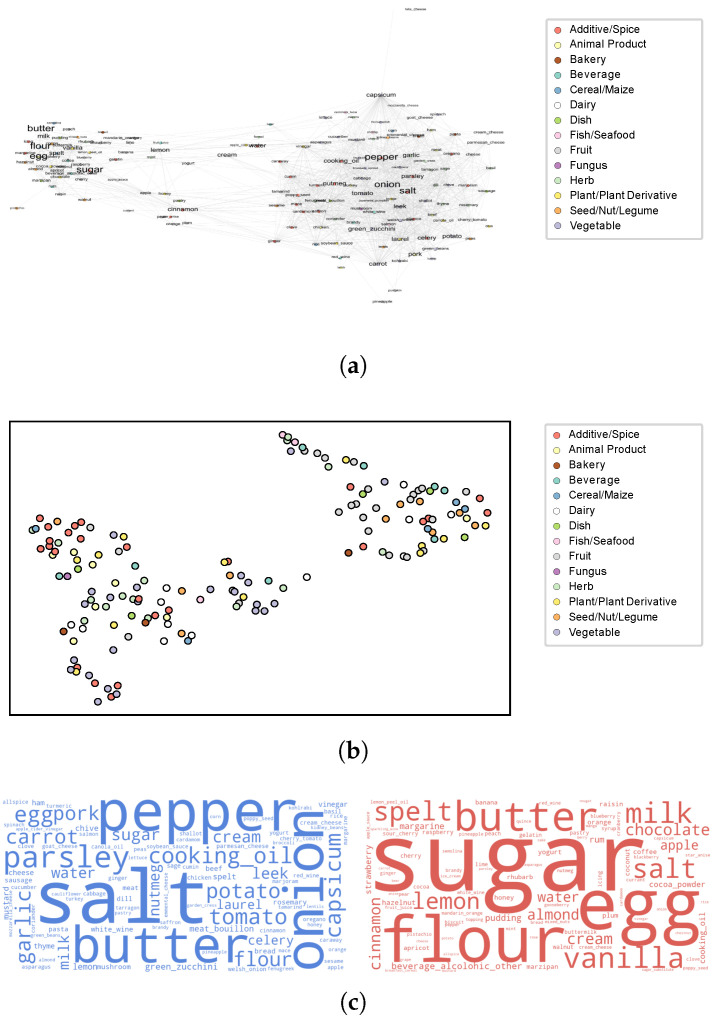
Subcommunities of savory/sweet recipes in appreciated and less appreciated recipes from *Kochbar*. (**a**) Ingredient complement network of appreciated and less appreciated recipes from *Kochbar*. (**b**) UMAP of Node2Vec embeddings generated from the network. (**c**) Word clouds of savory (**left**) and sweet (**right**) recipe ingredients in appreciated and less appreciated recipes from *Kochbar*.

Inspired by Teng’s work [62], we built ingredient complement networks for appreciated and less appreciated recipes from each recipe portal. We show the network building based on the *Xiachufang* recipes in Figure 6a. The network reveals clear subcommunities of savory and sweet recipes. Using Node2vec, we transformed ingredients in these networks into 128-dimensional vectors, which were visualized in 2D space using UMAP (Figure 6b). The vectors were then clustered using K-means, with *k* being set to 2. We illustrated the key ingredients in appreciated and less appreciated recipes using TF-IDF-weighted word clouds in Figure 6c. The clustering results clearly distinguish savory and sweet recipes. Savory recipes commonly feature salt and its complements, such as soybean sauce, garlic, and ginger in Chinese recipes. Sweet recipes, in contrast, emphasize sugar and its companions, including butter, eggs, and flour. Similar patterns were observed in the US and German portals; please see the ingredient networks (Figure A1a and Figure A2a), the UMAP of Node2Ved embeddings (Figure A1b and Figure A2b) and the corresponding word clouds (Figure A1c and Figure A2c) of the recipes from *Allrecipes* and *Kochbar* in Appendix C.

**Figure 7:**
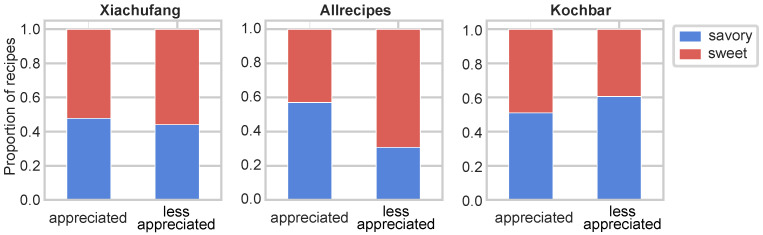
The proportion of savory and sweet recipes in appreciated and less appreciated recipes in each recipe portal.

We then analyzed the proportion of savory and sweet recipes in the appreciated and less appreciated categories across three recipe portals (Figure 7) to investigate the cross-cultural flavor preferences. In the Chinese portal, sweet and savory recipes were nearly equally distributed. *Allrecipes* showed a distinct preference, with savory recipes comprising 60% of appreciated recipes and sweet recipes dominating (75%) the less appreciated group. A contrasting trend in flavor preferences is displayed in *Kochbar*, where savory recipes make up 60% of the less appreciated recipes. These findings suggest nuanced cultural differences in flavor preferences: US user appear to favor sweet recipes less, while German users show a reduced preference for savory dishes. This finding aligns with the patterns observed by the algorithms, where classifiers from *Allrecipes* and *Kochbar* did not perform well on each others’ recipe collections.

However, it is important to note that we did not find a strong signal supporting the relatively superior performance of the classifiers from *Xiachufang* and *Allrecipes* on each others’ collections.

#### 3.3.2. Preferences for Sweet and Non-Sweet Flavors Across Cultures

**Figure 8:**
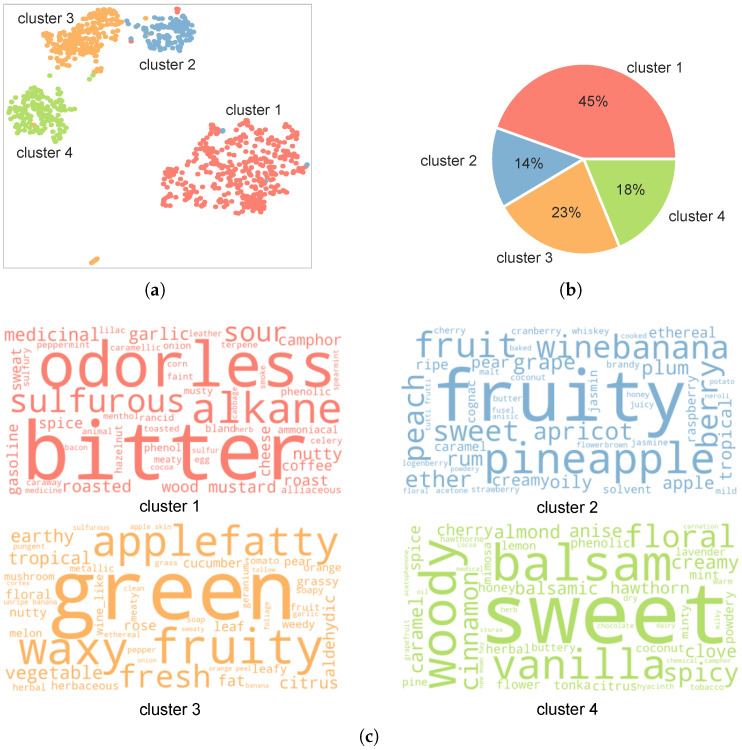
Clusters and flavor profiles of the flavor compounds. (**a**) The four clusters of the flavor compounds. (**b**) The proportions of the flavor compounds in each cluster. (**c**) Word clouds of the flavor compounds in each cluster.

Flavor profiles describe flavor compounds in a semantic fashion. Based on their relationship (see Figure 1), we represented each flavor compound with its corresponding flavor profiles. We applied K-means clustering to group flavor compounds with similar descriptions, resulting in four distinct clusters, as depicted in Figure 8a. Figure 8b displays the proportion of flavor compounds in each cluster. Approximately half the compounds were in cluster 1, with the remainder distributed across three additional clusters. To further understand the flavor compounds in each culture, we selected the 50 representative flavor profiles of the flavor compounds and visualized them, as shown in Figure 8c. The flavor compounds in cluster 1 are predominantly characterized by bitter and odorless profiles, while other clusters feature fruity and sweet profiles. We subsequently categorized flavor compounds into non-sweet (Cluster 1, 45%) and sweet (Clusters 2–4, 55%) groups to establish a baseline for comparing flavor preferences across Chinese, US, and German food cultures.

**Figure 9:**
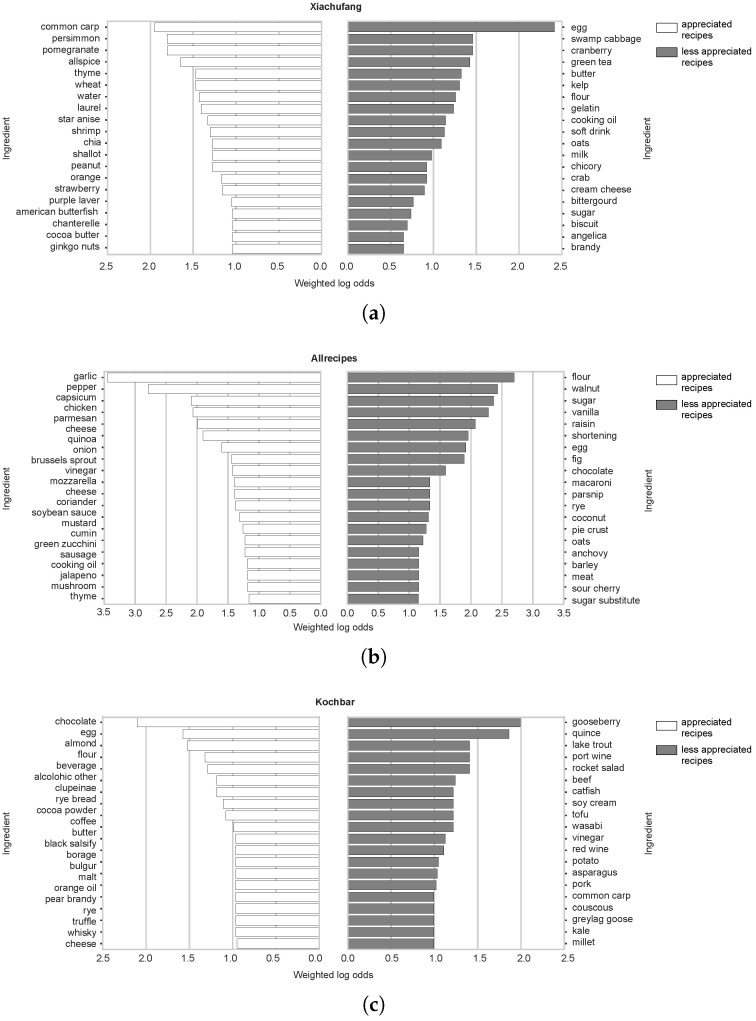
The distinctive ingredients of appreciated and less appreciated recipes from (**a**) *Xiachufang*, (**b**) *Allrecipes*, and (**c**) *Kochbar*.

Appreciated and less appreciated recipes from each recipe portal were represented by their top 50 distinctive ingredients. For improved visualization, we display only the top 20 distinctive ingredients for each group in Figure 9a–c. These distinctive ingredients indicate similar trends to those revealed by the ingredient complement networks. For instance, in the appreciated recipes from *Allrecipes*, ingredients from the savory cluster (e.g., garlic, pepper) are more prevalent, whereas less appreciated recipes contain more ingredients from the sweet cluster (e.g., flour, sugar). Conversely, in the *Kochbar* collection, ingredients such as chocolate and almond from the sweet cluster are distinctive in appreciated recipes, while less appreciated recipes feature more ingredients from savory recipes, such as lake trout and beef. These findings suggest that savory foods are more likely to be appreciated by US users, whereas sweet foods might be preferred by German users, indicating contrasting flavor preferences between the two cultures. The flavor description system constructed in this analysis further supports these findings, as shown in Table 3.

Table 3 presents the ratio of non-sweet and sweet flavor compounds found in the distinctive ingredients of appreciated and less appreciated recipes in *Xiachufang*, *Allrecipes*, and *Kochbar*. In *Xiachufang*, the non-sweet to sweet ratio in both appreciated and less appreciated recipes closely aligns with the baseline ratio (0.45:0.55) derived from the cluster analysis. However, differences are demonstrated in *Allrecipes* and *Kochbar*. In *Allrecipes*, appreciated recipes have a slightly higher proportion of non-sweet compounds (0.47) and a lower proportion of sweet compounds (0.53) compared to the baseline. Conversely, in *Kochbar*, appreciated recipes show a lower proportion of non-sweet compounds (0.42) and a higher proportion of sweet compounds (0.58). These findings align with algorithmic patterns, highlighting distinct food preferences in US and German cuisines that do not generalize across cultures.

## 4. Discussion

In this study, we explored the diversity of food flavors across cultures using online recipes from Chinese, US, and German portals. By linking ingredients to their corresponding flavor compounds, we created models to capture flavor information in these recipes. Our machine learning models, trained on these flavor vectors, successfully differentiated recipes from different cultural backgrounds, indicating tangible flavor differences across cultures. Additionally, our models demonstrated some success in predicting food preferences within each culture, although the task proved challenging based on the achieved accuracy. Moreover, by predicting the flavor preferences of one culture using models trained on another, although the accuracy was only slight above chance (baseline = 50%), we uncovered a signal of shared preferences across cultures, at least between Chinese and US users, which differed from those of German users. To the best of our knowledge, this is the first study in the food domain to explore such patterns using a data-driven approach based on flavor compounds. These algorithmic findings were supported by our two exploratory analyses. While these findings are promising, we acknowledge the exploratory nature of this work and recommend that future studies (e.g., consumer surveys, sensory experiments) are conducted to validate and deepen these insights. The theoretical and practical implications of our findings are discussed below.

### 4.1. Theoretical Implications

From a theoretical perspective, this work reveals a complex correlation between culture and food flavor. On the one hand, the performance (ACC = 0.77) of the models trained on flavor compounds for identifying recipe origins highlights distinct flavor differences across cultures. This aligns with the findings from Ahn’s work [9], which revealed flavor differences by means of food pairing theory. On the other hand, our transfer learning approach identified stable patterns in cross-cultural flavor preferences, which were also supported by the exploratory analyses. This is, to the best of our knowledge, the first study to reveal commonalities in food preferences across cultures in terms of flavor with large-scale online recipes. Drawing upon this, our work complements studies on cross-cultural sensory preferences examined through sensory evaluation [42,43] and consumer surveys [67,68] in two key ways. First, rather than focusing on small groups of panelists and participants, our approach utilizes tens of thousands of online recipes and user interaction data to uncover broader global dietary patterns. Second, instead of solely emphasizing how culture shapes diverse food preferences, we also identify underlying commonalities across cultures. We believe that the findings from our data-driven approach offer a novel perspective for understanding global food cultures.

In addition, compared to previous studies also based on online recipe data, our work shifts the focus from ingredient usage to flavor analysis. Drawing parallels with our previous work, which found stable patterns in food preferences in terms of visual aspects [41], we depart from traditional food studies focusing solely on ingredient composition. We argue that both visual appearance and flavor—key components of aesthetic aspects of food—offer valuable insights into cross-cultural food choices. Moreover, previous work displaying commonalities in a cross-cultural context primarily focused on geographically proximate regions [20,38]. Our study extends beyond geographical limitations to include three distinct cultures—China, the US, and Germany—demonstrating that stable patterns in food preferences can also be observed across more distantly related cultures.

### 4.2. Practical Implications

Our study primarily carries implications for the development of food recommender systems. Firstly, recognizing the differences in food flavors underscores the necessity of considering culture as a crucial contextual factor in personalized food recommender systems. Moreover, identifying commonalities in cross-cultural flavor preferences introduces a fresh perspective for cross-cultural food recommendations. Our results suggest the feasibility of recommending culturally appealing foods to users from diverse cultural backgrounds based on flavor information. This highlights the potential of flavor information in facilitating acceptable food recommendations for individuals engaged in cross-cultural interactions. Previous work has proven that taking flavor into consideration significantly enhances the performance of food recommender systems [69]. Building on our findings and insights from previous research, we propose several potential ways to integrate culture-related flavor preferences into food recommender systems:

- **User Profiling Based on Cultural Preferences:** The recommender system could incorporate user profiling that considers cultural background and flavor preferences. By asking users for their cultural backgrounds or inferring preferences based on their recipe interactions, this approach enables better alignment between users and food ontology [70], as well as recommend recipes that align with regional flavor profiles.
- **Flavor-Based Collaborative Filtering:** Collaborative filtering is a widely used approach in food recommender systems [71]. Previous studies have primarily clustered users’ preferences based on ingredient similarity [72,73,74]. Our findings suggest a new possibility of recommending food based on shared cultural backgrounds or flavor preferences, allowing users to receive personalized suggestions based on the choices of others with similar tastes.
- **Incorporating Contextual Factors:** Contextual information has been proposed to enhance the performance of food recommender systems [75,76,77]. Our findings suggest that integrating cultural factors, including regional food availability and traditional meal structures (e.g., the balance of flavors in different cuisines), could guide the recommendations to align better with users’ expectations based on their cultural preferences.

Our study also carries practical implications for recipe representation with flavor information. We show the effectiveness of modeling flavor with flavor compounds, offering a deeper understanding of flavor nuances compared to previous approaches like using macronutrients such as sodium content and sugar [69]. Moreover, flavor compound vectors show promise in predicting food preferences, aligning with previous suggestions that incorporating flavor information can enhance the performance of food recommender systems [69]. Our results highlight the suitability of TF-IDF vectors over Word2Vec embeddings for representing recipes in food preference prediction tasks. This may be due to the relatively small vocabulary size and our processing of the order of ingredient lists, which may limit the effectiveness of Word2Vec training. Future work should explore alternative embedding strategies, such as fine-tuning pre-trained models on larger food-related corpora or incorporating structural and contextual information to enhance representation quality.

We also demonstrate the potential of Node2Vec for recipe representation in this study. Although these node features were not directly involved in prediction tasks, applying unsupervised learning algorithms showed promising results in describing recipe flavor. This initial attempt at using node embeddings in the food domain aligns with recent work by Park et al. [63], who applied Node2Vec for food recommendation based on relationships between ingredients and flavor compounds. This suggests that node embeddings would be applicable in developing food recommender systems, with culture potentially serving as a valuable node for embedding in graphs.

Furthermore, our work facilitates the description of flavors. Our efforts aimed to make flavor information, particularly flavor compounds, more interpretable through their corresponding flavor profiles. While this investigation is preliminary computational, further analysis, such as evaluating the description systems through human perception and judgments, is necessary to assess their applicability in real-world scenarios.

### 4.3. Limitations and Future Directions

We recognize that our study leaves several questions unanswered. We acknowledge that some of our findings may be influenced by biases inherent in applying the data-driven methods on data derived from online sources.

First, in one of our exploratory analyses, we clustered recipes into savory and sweet groups by means of ingredient complement networks according to the approach described by [62], in which ingredients that co-occurred in the same recipe were considered complementary. However, given the user-generated nature of online recipes, the relationship between co-occurrence and complementarity was inferred based on user behavior rather than direct sensory validation. To the best of our knowledge, this assumption has not been explicitly validated through expert validation or sensory analysis. It was observed that the savory group contained primarily main courses, such as meatloaf and stir-fries, whereas the sweet group comprised more desserts like cakes and cookies. This raises the question “*Can we accurately classify savory and sweet recipes when limited to main dishes*?” If so, we must determine whether differences in preference for savory and sweet flavors persist between US and German users.

In addition, our exploratory analyses suggest that *Allrecipes* users tend to have a relatively low appreciation for sweet recipes. This challenges the common belief that US individuals consume excessive sweet food, raising potential public health concerns [78]. This may be attributed to the temporal limitations of our dataset, since the data were collected several years ago and span different time periods (e.g., 2015 and 2018), which may limit their reflection of current dietary preferences. Future research incorporating more recent data is encouraged to examine the persistence of these patterns over time. Another possible explanation for this finding might be the inconsistency in appreciation metrics across platforms. Since multiple metrics are available in the recipe portals, particularly in *Allrecipes* and *Kochbar*, additional investigation is needed to explore the correlations between metrics indicating users’ recipe preferences, such as the number of bookmarks, ratings, comments, and the sentiments expressed in their comments. Trattner et al. [53] have highlighted a significant correlation between the number of comments and ratings that the recipes on *Allrecipes* have received within a day/week/month/year. However, the number of bookmarks demonstrates only a limited correlation with these other metrics [79]. Investigating the relationships among these metrics is crucial to understanding users’ bookmarking behavior and to what extent it accurately reflects their recipe preferences. It is important to determine if there is a scenario where “*Users rate sweet recipes more often, leave more positive comments, but bookmark them less frequently*”. Addressing these issues would enhance our understanding of US users’ flavor preferences for non-sweet and sweet recipes, ultimately improving the accuracy of flavor-based food recommendations.

We also found that the proposed flavor categorization in our work, with an ingredient complement network and the semantic clustering of flavor compounds, offers only a coarse classification. To be specific, our current data-driven approach is limited to distinguishing between sweet and non-sweet/savory flavors. The other basic flavors perceived by humans, such as sour, bitter, and umami, remain unexplored in this study due to limitations in the available data and methodology. To the best of our knowledge, this work represents the first initial exploratory effort to describe flavor preferences through statistical techniques. We believe it lays useful groundwork for future refinement and sensory validation, which could incorporate a broader and more nuanced range of flavor perceptions.

Taken together, these limitations highlight the need for further validation of our findings. Our study is based primarily on user-generated recipes and interaction data, which may not fully capture the diversity of population-level dietary behaviors. In future work, we aim to collaborate closely with food scientists, particularly experts in sensory evaluation and consumer research. For example, cross-cultural sensory tests with participants from different cultural backgrounds could help confirm whether flavor preferences identified through online recipe analysis are consistent with the sensory perceptions of individuals from those regions. These tests could begin with participants from the same cultural backgrounds as in this work, namely China, the US and Germany. Follow-up qualitative approaches, such as semi-structured interviews and focus groups, would be valuable for uncovering the mechanisms behind flavor preference formation. These approaches could also complement our current data-driven classification of flavor (i.e., savory/non-sweet vs. sweet) by not only validating these preferences across cultures but also providing richer insights into other basic taste categories, such as sour, bitter, and umami. Furthermore, with the goal of developing culturally aware food recommender systems, we aim to integrate cross-cultural flavor preferences into personalized food recommendation models, enhancing their ability to provide more contextually and culturally relevant food suggestions. Potential methods for future work, either by us or our collaborators, include user profiling based on cultural preferences, flavor-based collaborative filtering, and incorporating contextual factors, as detailed in Section 4.2.

## 5. Conclusions

By applying machine learning algorithms and conducting subsequent exploratory analyses, this study has yielded three primary findings. First, we identified distinct flavor differences across Chinese, US, and German cultures. Second, although challenging, predicting flavor preferences within each culture is possible. Third, we observed a preliminary signal of stable patterns in cross-cultural flavor preferences, particularly between Chinese and US users. Our subsequent data-driven exploratory analyses contribute to supporting the patterns revealed by the algorithms.

Taken together, these findings have both theoretical and practical implications. Theoretically, this study deepens our understanding of cross-cultural food preferences. In contrast to previous work that primarily highlights cultural differences, our findings suggest the existence of underlying stable patterns in flavor preferences across cultures. Additionally, this work, along with our previous work focusing on the visual aspects of food, further underscores the crucial role sensory inputs play in shaping dietary choices in different cultural backgrounds. Looking ahead, we see promising practical implications for food recommender systems. Addressing the challenges of food recommendation in cross-cultural scenarios, our study proposes a new perspective that incorporates cross-cultural preference with flavor preferences. This could potentially lead to culturally sensitive and contextually appropriate recommendations. Furthermore, our findings contribute to enhancing recipe representation through flavor information, rather than relying solely on the food pairing theory in previous work, paving the way for further investigations into flavor-related aspects in the field of food studies.

However, it is important to acknowledge that our work is exploratory in nature. Given that the analysis is primarily based on online recipe data collected several years ago, future work should incorporate more recent and comprehensive datasets to examine the temporal robustness of the observed patterns. In addition, our findings, generated through online user-generated data and data-driven approaches, should be validated through controlled sensory studies and consumer surveys to ensure robustness. Future research will involve collaborations with sensory scientists and experts in consumer behavior to further substantiate and expand upon these insights.

In summary, this study represents a step toward a more comprehensive understanding of the sensory dimensions of food preferences across cultures. We are optimistic that our findings will contribute to the development of food recommender systems and further exploration of flavor information.

## Figures and Tables

**Table 1:** The results of predicting which portals the recipes belong to based on ingredient and flavor compound vectors (TF-IDF and Word2Vec).

Features	Accuracy		
	NB	LOG	RF
Ingredient (TF-IDF)	0.77	**0.81**	0.73
Flavor Compounds (TF-IDF)	0.65	**0.77**	0.74
Ingredient (Word2Vec)	0.56	0.73	0.72
Flavor Compounds (Word2Vec)	0.35	0.66	0.70

Best-performing scores for each classifier are bold. *NB* = Naive Bayes; *LOG* = Logistic Regression; *RF* = Random Forest.

**Table 2:** Results of identifying appreciated and less appreciated recipes in each recipe portal with ingredient and flavor compound vectors (TF-IDF and Word2Vec).

Features	Xiachufang			Allrecipes			Kochbar		
	Accuracy			Accuracy			Accuracy		
	NB	LOG	RF	NB	LOG	RF	NB	LOG	RF
Ingredient (TF-IDF)	0.62	0.65	**0.67**	0.67	**0.70**	0.68	0.62	**0.63**	0.61
Flavour Compounds (TF-IDF)	0.60	0.61	**0.66**	0.64	0.66	**0.67**	0.59	**0.65**	0.64
Ingredient (Word2Vec)	0.60	0.64	0.64	0.58	0.65	0.67	0.54	0.57	0.54
Flavour Compounds (Word2Vec)	0.56	0.61	0.63	0.51	0.65	0.64	0.48	0.61	0.60

Best-performing scores for each classifier are bold. *NB* = Naive Bayes; *LOG* = Logistic Regression; *RF* = Random Forest.

**Table 3:** The ratio of non-sweet and sweet flavor compounds in the appreciated and less appreciated recipes in each recipe collection.

	Xiachufang		Allrecipes		Kochbar	
	**Non-Sweet** **Flavor Compounds**	**Sweet** **Flavor Compounds**	**Non-Sweet** **Flavor Compounds**	**Sweet** **Flavor Compounds**	**Non-Sweet** **Flavor Compounds**	**Sweet** **Flavor Compounds**
**Appreciated recipes**	0.45	0.55	0.47^+^	0.53^−^	0.42^−^	0.58^+^
**Less appreciated recipes**	0.46	0.54	0.45	0.55	0.45	0.55

^+^ higher than the baseline, ^−^ lower than the baseline.

## Data Availability

The original contributions presented in the study are included in the article, further inquiries can be directed to the corresponding author.

## Appendix A

### Appendix A.1. Multilingual Recipe Data Preprocessing

We investigated both the ingredients contained within recipes and the flavor information of recipes. In order to extract the flavor information, we mapped the ingredients in the online recipes to their corresponding flavor compounds according to FlavorDB [49], which contains 936 ingredient entities along with their associated 2254 flavor molecules. The following steps were taken to preprocess and clean the data:

- ***Removing non-ingredients terms:*** The majority of online recipes were composed in free-text format, often containing non-ingredient terms such as punctuation, and emojis, as well as kitchen/eating utensils (e.g., aluminum foil, bowl), amounts, and units (e.g., 2 L, 1/4 pounds etc.). In our dataset, we retained only the ingredients, removing all non-ingredient terms.
- ***Translating the ingredient lists:*** In this step, we translated the ingredient lists of online recipes in Chinese and German to English. To facilitate this process, we initiated translation requests to Google Cloud Translation API v2 (https://cloud.google.com/translate/docs/basic/translating-text, accessed on 14 April 2025), allowing for automated translation. Although this automated approach successfully translated the bulk of the ingredient lists, it is important to note that there were instances where certain ingredients were inaccurately translated. To address this, we conducted a manual review and made necessary correctiosn to the translations.
- ***Removing the “stop words”:*** In this study, “stop words” refer to frequently occurring non-noun words in online recipes, often adverbs, adjectives, or past tense verbs, which can interfere with the mapping process. For instance, “very ripe bananas” or “chopped onion” contain “stop words” such “very”, “ripe”, and “chopped”. To identify these “stop words”, we employed the Stanford NLP Group’s Part-of-Speech Tagger (POS Tagger), which successfully recognized most non-noun words as “stop words”. However, exceptions existed, such as the use of certain nouns such as “slice” in “bacon in slice”, “sticks” in “cinnamon sticks”, and “chunks” in “pineapple chunks”, which needed to be removed. However, some non-noun words like “green” in “green beans” and “sweet” in “sweet potato” could not be removed without altering the ingredient meaning and were handled manually.
- ***Mapping ingredients to FlavorDB entities:*** After the processes above, there were ingredients that could be mapped to entities in FlavorDB directly. Nevertheless, to maximize the coverage of ingredient mapping, a substantial portion of manual work was undertaken. Here, we outline several common situations encountered during this manual process and our corresponding approaches. We first standardized the ingredients according to the entity granularity within FlavorDB. For example, the variations in ingredients (e.g., apple variations *Goldparmäne*, *Boskop*) were normalized to their broader categories. Similarly, components of poultry/animal products, such as *chicken breast/wings*, *egg yolk/white*, were unified with their respective origins. In addition, extracts or products used in recipes, such as *orange juice* and *anise powder*, were aligned with their fundamental components. Apart from the aforementioned ingredients, there were also food products or mixed ingredients, such as *strawberry yogurt*. In such cases, we modeled *strawberry yogurt* as flavor compounds of strawberry and yogurt, as *strawberry yogurt* has the flavors of both strawberry and yogurt. This modeling approach was also extended to ingredients such as *vanilla pudding* and *chocolate cream*. Similarly, we applied this process to mixed spices like *Herbes de Provence* and *Suppengrün*. We divided these into individual ingredients based on authority recipes or appreciated recipes from the recipe portals.

After the data cleaning and processing, approximately 34% of ingredients in *Xiachufang* recipes, 43% of ingredients in *Allrecipes* recipes, and 19% of ingredients in *Kochbar* recipes were mapped to entities in FlavorDB, as well as their corresponding flavor compounds. The numbers of raw terms in the online recipes, cleaned and processed ingredients, and ingredients with flavor compounds are shown in Table A1.

**Table A1:** The number of terms (before preprocessing) and the number of ingredients mapped to FlavorDB ingredient names (after preprocessing) in the recipes from each recipe portal.

Origin	Num. Raw Termsin Recipes ^1^	Num. Processed Ingredients in Recipes ^2^	Num. Ingredients with Flavor Compounds
Xiachufang	10,584	973	331
Allrecipes	30,333	824	356
Kochbar	42,022	2084	401

^1^ The column *Num. Raw Terms in Recipes* refers to the number of instances of text split with ‘,’ in the raw recipes, including the non-ingredients terms. ^2^ The column *Num. Processed Ingredients in Recipes* refers to the number of ingredients that were processed.

### Appendix A.2. Recipe Representation with Word2Vec

We present the fine-tuning of the Word2Vec hyperparameters for representing ingredients and flavor compounds below:

- *size*: This parameter determines the dimension of vectors for representing each word. We applied the default value 100 in this work, so that each ingredient was represented by a 100-dimensional vector.
- *min_count*: This parameter regulates the minimum frequency of words that can be input into the model. To be specific, if the default value of *min_count* is 5, then only words that occur more than or equal to five times would be input into the model. In this work, we set the value to 1, in order to represent every ingredient and flavor compound in the dataset.
- *window*: This parameter determines how many words around the target word are taken into consideration when the model is training. We used the default value of 5 in this work, which means that for each ingredient/flavor compound in the recipe, the model learns its embeddings by considering five ingredients/flavor compounds before and after it.

## Appendix B. Ingredient Representation with Node2Vec

We display the fine-tuning of Node2Vec for representing the ingredients below:

- *dimensions*: This parameter refers to the dimensions of the generated node embeddings. We chose the default value 128, so that each ingredient in the networks was represented as a 128-dimensional vector.
- *num_walks*: This parameter determines the number of random walks per node. The default value of the parameter is 10. However, optimal parameter values may vary across different scenarios [64]; thus, we undertook a series of experiments to evaluate various values for this parameter. Specifically, during the generation of the node embeddings for the ingredients in the complement networks of recipes in the *Xiachufang*, *Allrecipes*, and *Kochbar* collections, values from 10 to 25 were examined. To effectively analyze the resulting node embeddings, we applied UMAP to display these in a two-dimensional layout. The insights gained from these visualizations guided our determination of which parameter value most effectively reflects the underlying structure of the networks.
- *walk_length*: This parameter indicates the number of nodes traversed in each random walk. The default value is 80. We employed a similar approach to determine the value for this parameter to that which we used for *num_walk*. A range from 15 to 25 was tested to ascertain the optimal settings for this parameter.
- *p* and *q: p* refers to the *Return* hyperparameter, determining the possibility of revisiting a former node in a walk after visiting a node. Setting a higher value of *p* ensures a lower chance of revisiting a node. The default value of *p* is 1, and we set it to 2 to enable a broader exploration of the graph. *q* is the *Inout* hyperparameter, determining the probability of exploring undiscovered nodes. A high value of *q* (*q* > 1) allows a random walk towards the closer neighbors of the previous nodes, which leads to a local view of the network with respect to the start node in the walk and approximate *Breadth first Sampling* (*BFS*) behavior. In contrast, a lower value of *q* (*q* < 1) encourages the walk to explore the network with *Depth first Sampling* (*DFS*) behavior and offers a macro-view of the network. The default *q* is 1. In this work, we set *q* to 0.25 in order to obtain a macro-view of the ingredient complement networks.

## Appendix C

Below, we present the ingredient complement networks constructed from online recipes on Allrecipes (Figure A1) and Kochbar (Figure A2), along with the corresponding UMAP visualizations of Node2Vec embeddings in 2D space and word clouds of ingredients in sweet and savory recipes.
